# Supplementary material for: A prospective diagnostic evaluation of accuracy of self-taken and healthcare worker-taken swabs for rapid COVID-19 testing
Source: PLoS One. 2022 Jun 30;17(6):e0270715. doi: 10.1371/journal.pone.0270715 (PMC9246218; doi:10.1371/journal.pone.0270715)

**Appendix:**


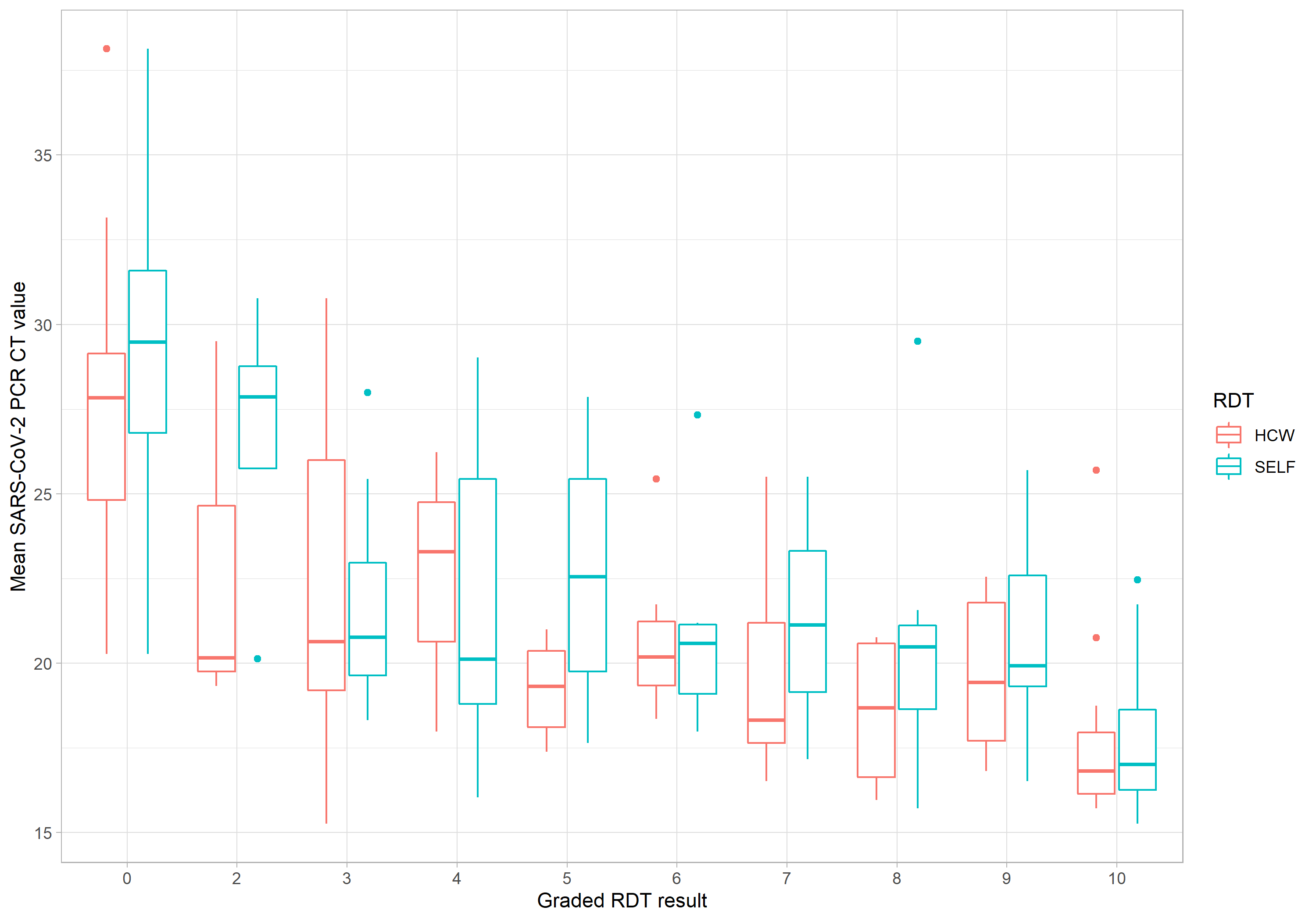
 A box plot comparing self-taken and healthcare worker taken swabs tested by Covios® RDT by mean PCR CT value.

Self-taken and HCW taken Covios® Ag RDT results by RT-PCR CT range.


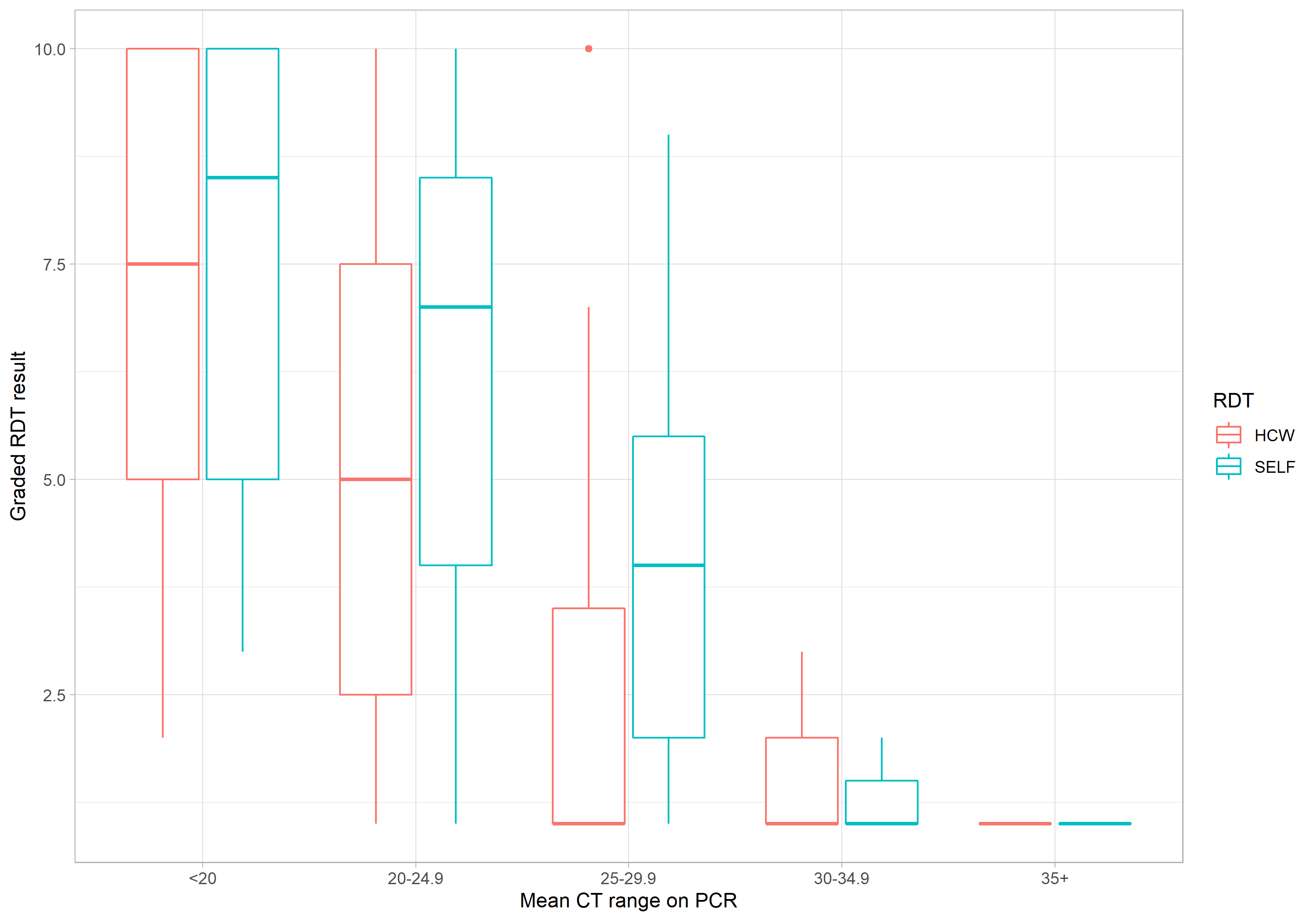

Supplement: S1 Appendix — (DOCX) [file pone.0270715.s001.docx]
