# Supplementary material for: A prospective diagnostic evaluation of accuracy of self-taken and healthcare worker-taken swabs for rapid COVID-19 testing
Source: PLoS One. 2022 Jun 30;17(6):e0270715. doi: 10.1371/journal.pone.0270715 (PMC9246218; doi:10.1371/journal.pone.0270715)
Supplement: S1 Table — (DOCX) [file pone.0270715.s002.docx]

| Participant | Age | Gender | Days of symptoms | Shortness of breath | Cough | Fever | Chest pain | Sore throat | Confusion | Rash | Loss of smell | Loss of taste | Abdominal pain | Vomiting | Diarrhoea | Headache | Tiredness/Fatigue | Tight chest | Other | Other symptom | Ethnicity | Vaccine | 1st dose | 2nd dose | Self RDT result | Self - Reader 1/Reader2 | HCW RDT result | HCW - Reader1/Reader2 | PCR result | N gene | ORF1ab | S gene | Mean CT value |
| --- | --- | --- | --- | --- | --- | --- | --- | --- | --- | --- | --- | --- | --- | --- | --- | --- | --- | --- | --- | --- | --- | --- | --- | --- | --- | --- | --- | --- | --- | --- | --- | --- | --- |
| 1 | 36 | Female | 1 | No | Yes | No | No | No | No | No | No | No | No | No | No | No | No | No | No |  | White British | Yes | Yes | Yes | Negative | 0/0 | Negative | 0/0 | Negative | 0 | 0 | 0 | 0 |
| 2 | 37 | Female | 1 | No | Yes | No | No | No | No | No | No | No | No | No | No | No | No | No | No |  | White British | No | NA | NA | Negative | 0/0 | Negative | 0/0 | Negative | 0 | 0 | 0 | 0 |
| 3 | 38 | Female | 4 | No | No | No | No | No | No | No | Yes | Yes | No | No | No | No | No | No | No |  | White British | Yes | Yes | Yes | Negative | 0/0 | Negative | 0/0 | Negative | 0 | 0 | 0 | 0 |
| 4 | 51 | Female | 4 | No | No | No | No | Yes | No | No | Yes | Yes | No | No | No | No | No | No | Yes | Myalgia | White British | Yes | Yes | No | Negative | 0/0 | Negative | 0/0 | Negative | 0 | 0 | 0 | 0 |
| 5 | 76 | Female | 2 | No | Yes | No | No | No | No | No | No | No | No | No | No | Yes | No | No | Yes | Myalgia | White British | Yes | Yes | No | Negative | 0/0 | Negative | 0/0 | Negative | 0 | 0 | 0 | 0 |
| 6 | 40 | Female | 7 | No | Yes | Yes | Yes | No | No | No | No | No | No | No | No | Yes | No | No | Yes | Aches | White British | Yes | Yes | No | Positive | 4/4 | Positive | 10/10 | Positive | 15.91 | 16.16 | 0 | 16.04 |
| 7 | 45 | Female | 2 | No | Yes | Yes | No | No | No | No | No | No | No | No | No | No | No | No | Yes | Swollen glands | Other | No | NA | NA | Positive | 8/8 | Positive | 5/5 | Positive | 18.43 | 18.39 | 0 | 18.41 |
| 8 | 69 | Female | 2 | No | Yes | No | No | Yes | No | No | No | No | No | No | No | No | No | No | No |  | Other | Yes | Yes | No | Negative | 0/0 | Negative | 0/0 | Negative | 0 | 0 | 0 | 0 |
| 9 | 29 | Female | 33 | No | Yes | No | No | No | No | No | No | No | No | No | No | No | No | No | No |  | White British | No | NA | NA | Negative | 0/0 | Negative | 0/0 | Negative | 0 | 0 | 0 | 0 |
| 10 | 69 | Male | 1 | No | No | No | No | Yes | No | No | No | No | No | No | No | Yes | Yes | No | No |  | White British | Yes | Yes | No | Negative | 0/0 | Negative | 0/0 | Negative | 0 | 0 | 0 | 0 |
| 11 | 32 | Female | 1 | No | Yes | No | No | No | No | No | Yes | Yes | No | No | No | No | No | No | Yes | Cold | White British | No | NA | NA | Negative | 0/0 | Negative | 0/0 | Negative | 0 | 0 | 0 | 0 |
| 12 | 52 | Male | 2 | No | Yes | No | No | No | No | No | No | No | No | No | No | No | No | No | Yes | Aches | White British | Yes | Yes | No | Negative | 0/0 | Negative | 0/0 | Negative | 0 | 0 | 0 | 0 |
| 13 | 31 | Female | 4 | No | Yes | Yes | No | No | No | No | No | No | No | No | No | No | No | No | No |  | White British | No | NA | NA | Negative | 0/0 | Negative | 0/0 | Negative | 0 | 0 | 0 | 0 |
| 14 | 36 | Male | 3 | No | Yes | No | No | No | No | No | No | No | No | No | No | No | No | No | No |  | White British | No | NA | NA | Negative | 0/0 | Negative | 0/0 | Negative | 0 | 0 | 0 | 0 |
| 15 | 40 | Male | 0 | No | No | Yes | No | No | No | No | No | No | No | No | No | Yes | No | No | Yes | Dry throat | White British | No | NA | NA | Negative | 0/0 | Negative | 0/0 | Negative | 0 | 0 | 0 | 0 |
| 16 | 54 | Male | 5 | No | Yes | Yes | No | No | No | No | No | No | No | No | No | Yes | No | No | Yes | Nausea | White British | Yes | Yes | No | Positive | 10/10 | Positive | 10/10 | Positive | 16.1 | 16.41 | 0 | 16.26 |
| 17 | 34 | Female | 3 | No | Yes | No | No | No | No | No | No | No | No | No | No | No | No | No | No |  | White British | Yes | Yes | No | Negative | 0/0 | Negative | 0/0 | Negative | 0 | 0 | 0 | 0 |
| 18 | 50 | Female | 2 | No | No | Yes | No | No | No | No | No | Yes | No | No | No | No | No | No | No |  | White British | Yes | Yes | No | Negative | 0/0 | Negative | 0/0 | Negative | 0 | 0 | 0 | 0 |
| 19 | 32 | Female | 4 | No | No | No | No | Yes | No | No | No | No | No | No | No | Yes | Yes | No | No |  | White British | No | NA | NA | Negative | 0/0 | Negative | 0/0 | Negative | 0 | 0 | 0 | 0 |
| 20 | 61 | Female | 6 | No | Yes | No | No | No | No | No | No | No | No | Yes | No | No | No | No | No |  | White British | Yes | Yes | No | Negative | 0/0 | Negative | 0/0 | Negative | 0 | 0 | 0 | 0 |
| 21 | 59 | Female | 5 | No | Yes | No | No | No | No | No | No | No | No | No | No | Yes | No | No | No |  | White British | Yes | Yes | No | Negative | 0/0 | Negative | 0/0 | Negative | 0 | 0 | 0 | 0 |
| 22 | 59 | Male | 0 | No | No | No | No | Yes | No | No | No | No | No | No | No | Yes | No | No | No |  | White British | Yes | Yes | No | Negative | 0/0 | Negative | 0/0 | Negative | 0 | 0 | 0 | 0 |
| 23 | 35 | Female | 0 | No | No | Yes | No | No | No | No | No | No | No | No | No | No | No | No | No |  | White British | No | NA | NA | Negative | 0/0 | Negative | 0/0 | Negative | 0 | 0 | 0 | 0 |
| 24 | 20 | Male | 2 | No | Yes | Yes | No | No | No | No | No | No | No | No | No | No | No | No | Yes | Change in taste | Other | No | NA | NA | Negative | 0/0 | Negative | 0/0 | Negative | 0 | 0 | 0 | 0 |
| 25 | 52 | Male | 4 | No | Yes | No | No | No | No | No | No | No | No | No | No | No | No | No | No |  | White British | Yes | Yes | No | Negative | 0/0 | Negative | 0/0 | Negative | 0 | 0 | 0 | 0 |
| 26 | 59 | Male | 1 | No | Yes | No | No | No | No | No | No | No | No | No | No | Yes | No | No | Yes | Flu like symptoms | White British | Yes | Yes | No | Positive | 4/4 | Negative | 0/0 | Positive | 28.65 | 29.41 | 0 | 29.03 |
| 27 | 36 | Female | 6 | No | Yes | No | No | No | No | No | No | No | No | No | No | No | No | No | No |  | Other | No | NA | NA | Negative | 0/0 | Negative | 0/0 | Negative | 0 | 0 | 0 | 0 |
| 28 | 46 | Male | 2 | No | Yes | No | No | No | No | No | No | No | Yes | No | No | Yes | No | No | No |  | White British | Yes | Yes | No | Negative | 0/0 | Negative | 0/0 | Negative | 0 | 0 | 0 | 0 |
| 29 | 25 | Female | 1 | No | Yes | No | No | Yes | No | No | No | No | No | No | No | No | No | No | No |  | White British | No | NA | NA | Negative | 0/0 | Negative | 0/0 | Negative | 0 | 0 | 0 | 0 |
| 30 | 46 | Female | 12 | No | Yes | No | No | No | No | No | No | No | No | No | No | No | No | No | No |  | White British | Yes | Yes | No | Negative | 0/0 | Negative | 0/0 | Negative | 0 | 0 | 0 | 0 |
| 31 | 36 | Female | 1 | No | No | Yes | No | No | No | No | No | No | No | No | No | No | No | No | No |  | Other | No | NA | NA | Negative | 0/0 | Negative | 0/0 | Negative | 0 | 0 | 0 | 0 |
| 32 | 55 | Female | 11 | Yes | Yes | Yes | No | Yes | No | No | No | No | No | No | No | No | No | No | No |  | White British | No | NA | NA | Negative | 0/0 | Negative | 0/0 | Negative | 0 | 0 | 0 | 0 |
| 33 | 64 | Male | 2 | No | No | Yes | No | No | No | No | No | No | No | No | No | No | No | No | No |  | White British | Yes | Yes | No | Negative | 0/0 | Negative | 0/0 | Negative | 0 | 0 | 0 | 0 |
| 34 | 38 | Male | 1 | No | Yes | No | No | No | No | No | No | No | No | No | No | No | No | No | No |  | White British | No | NA | NA | Negative | 0/0 | Negative | 0/0 | Negative | 0 | 0 | 0 | 0 |
| 35 | 24 | Male | 1 | No | No | Yes | No | Yes | No | No | No | No | No | No | No | No | No | No | Yes | Body aches | White British | No | NA | NA | Negative | 0/0 | Negative | 0/0 | Negative | 0 | 0 | 0 | 0 |
| 36 | 56 | Female | 2 | No | Yes | No | No | No | No | No | No | No | No | No | No | Yes | No | No | No |  | White British | Yes | Yes | No | Negative | 0/0 | Negative | 0/0 | Negative | 0 | 0 | 0 | 0 |
| 37 | 52 | Male | 1 | No | No | Yes | No | No | No | No | No | No | No | No | No | Yes | No | No | No |  | White British | Yes | Yes | No | Negative | 0/0 | Negative | 0/0 | Negative | 0 | 0 | 0 | 0 |
| 38 | 34 | Male | 10 | No | Yes | No | No | Yes | No | No | No | No | No | No | No | No | No | No | No |  | White British | No | NA | NA | Negative | 0/0 | Positive | 2/2 | Negative | 0 | 0 | 0 | 0 |
| 39 | 29 | Female | 3 | No | No | No | No | Yes | No | No | No | No | No | No | No | Yes | No | No | Yes | Nausea | Other | Yes | Yes | Yes | Positive | 4/4 | Positive | 2/2 | Negative | 0 | 0 | 0 | 0 |
| 40 | 32 | Male | 2 | No | Yes | No | No | No | No | No | No | No | No | No | No | No | No | No | Yes | Body aches,hot and cold | White British | No | NA | NA | Negative | 0/0 | Negative | 0/0 | Negative | 0 | 0 | 0 | 0 |
| 41 | 27 | Male | 1 | No | Yes | No | No | Yes | No | No | No | No | No | No | No | No | No | No | Yes | Run down | Other | No | NA | NA | Negative | 0/0 | Negative | 0/0 | Negative | 0 | 0 | 0 | 0 |
| 42 | 24 | Female | 2 | No | Yes | Yes | No | Yes | No | No | No | No | No | No | No | No | No | No | No |  | White British | No | NA | NA | Negative | 0/0 | Negative | 0/0 | Negative | 0 | 0 | 0 | 0 |
| 43 | 44 | Male | 3 | No | Yes | Yes | No | No | No | No | No | No | No | No | No | No | No | No | No |  | White British | No | NA | NA | Negative | 0/0 | Negative | 0/0 | Negative | 0 | 0 | 0 | 0 |
| 44 | 36 | Female | 5 | No | Yes | Yes | No | No | No | No | No | No | No | No | No | No | No | No | Yes | Pains and aches | White British | No | NA | NA | Negative | 0/0 | Negative | 0/0 | Negative | 0 | 0 | 0 | 0 |
| 45 | 41 | Male | 1 | No | No | Yes | No | No | No | No | No | No | No | No | No | Yes | No | No | Yes | Chills/aches and pains | White British | No | NA | NA | Negative | 0/0 | Negative | 0/0 | Negative | 0 | 0 | 0 | 0 |
| 46 | 30 | Female | 1 | No | Yes | No | No | No | No | No | No | No | No | No | No | No | No | No | No |  | White British | No | NA | NA | Negative | 0/0 | Negative | 0/0 | Negative | 0 | 0 | 0 | 0 |
| 47 | 27 | Female | 2 | No | Yes | No | No | No | No | No | No | No | No | No | No | No | No | No | No |  | White British | Yes | Yes | No | Negative | 0/0 | Negative | 0/0 | Negative | 0 | 0 | 0 | 0 |
| 48 | 36 | Female | 3 | No | Yes | No | No | Yes | No | No | No | No | No | No | No | No | No | No | No |  | White British | No | NA | NA | Negative | 0/0 | Negative | 0/0 | Negative | 0 | 0 | 0 | 0 |
| 49 | 55 | Female | 10 | No | Yes | No | No | No | No | No | No | No | No | No | No | No | No | No | No |  | Other | No | NA | NA | Negative | 0/0 | Negative | 0/0 | Negative | 0 | 0 | 0 | 0 |
| 50 | 32 | Male | 3 | No | Yes | No | Yes | Yes | No | No | No | No | No | No | No | No | No | No | No |  | White British | No | NA | NA | Negative | 0/0 | Negative | 0/0 | Negative | 0 | 0 | 0 | 0 |
| 51 | 39 | Female | 2 | No | Yes | No | No | No | No | No | No | No | No | No | No | No | No | No | No |  | White British | No | NA | NA | Negative | 0/0 | Negative | 0/0 | Negative | 0 | 0 | 0 | 0 |
| 52 | 38 | Female | 7 | No | Yes | Yes | No | No | No | No | No | No | No | No | No | No | No | No | No |  | Other | Yes | Yes | No | Negative | 0/0 | Negative | 0/0 | Negative | 0 | 0 | 0 | 0 |
| 53 | 37 | Male | 1 | No | Yes | No | No | Yes | No | No | No | No | No | No | No | Yes | No | No | No |  | White British | No | NA | NA | Negative | 0/0 | Negative | 0/0 | Negative | 0 | 0 | 0 | 0 |
| 54 | 25 | Male | 2 | No | Yes | No | No | Yes | No | No | No | No | No | No | No | Yes | No | No | No |  | White British | Yes | Yes | No | Negative | 0/0 | Negative | 0/0 | Negative | 0 | 0 | 0 | 0 |
| 55 | 28 | Male | 4 | No | Yes | No | No | Yes | No | No | No | No | No | No | No | No | No | No | No |  | White British | No | NA | NA | Negative | 0/0 | Negative | 0/0 | Negative | 0 | 0 | 0 | 0 |
| 56 | 57 | Male | 2 | No | No | Yes | No | No | No | No | No | No | No | No | No | No | No | No | No |  | White British | Yes | Yes | No | Negative | 0/0 | Negative | 0/0 | Negative | 0 | 0 | 0 | 0 |
| 57 | 25 | Female | 2 | No | No | No | No | Yes | No | No | No | No | No | No | Yes | No | No | No | No |  | White British | No | NA | NA | Negative | 0/0 | Negative | 0/0 | Negative | 0 | 0 | 0 | 0 |
| 58 | 58 | Female | 2 | Yes | Yes | Yes | No | No | No | No | No | No | No | No | No | Yes | No | No | Yes | Sneezing | White British | Yes | Yes | Yes | Negative | 0/0 | Negative | 0/0 | Negative | 0 | 0 | 0 | 0 |
| 59 | 67 | Female | 5 | Yes | Yes | No | No | No | No | No | No | No | No | No | No | No | No | No | No |  | White British | Yes | Yes | Yes | Negative | 0/0 | Negative | 0/0 | Negative | 0 | 0 | 0 | 0 |
| 60 | 45 | Male | 3 | No | Yes | No | No | No | No | No | No | No | No | No | No | No | No | No | Yes | Cold | Other | Yes | Yes | No | Negative | 0/0 | Negative | 0/0 | Negative | 0 | 0 | 0 | 0 |
| 61 | 33 | Female | 3 | No | Yes | No | No | No | No | No | No | No | No | No | No | No | No | No | No |  | White British | No | NA | NA | Negative | 0/0 | Negative | 0/0 | Negative | 0 | 0 | 0 | 0 |
| 62 | 27 | Female | 1 | No | Yes | No | No | No | No | No | No | No | No | No | No | No | No | No | Yes | Flu-like symptoms | White British | Yes | Yes | No | Negative | 0/0 | Negative | 0/0 | Negative | 0 | 0 | 0 | 0 |
| 63 | 43 | Female | 4 | No | Yes | No | No | Yes | No | No | No | No | No | No | No | No | No | No | Yes | Lost voice | White British | Yes | Yes | Yes | Negative | 0/0 | Negative | 0/0 | Negative | 0 | 0 | 0 | 0 |
| 64 | 38 | Female | 2 | No | Yes | No | No | No | No | No | No | No | No | No | No | No | No | No | No |  | White British | No | NA | NA | Negative | 0/0 | Negative | 0/0 | Negative | 0 | 0 | 0 | 0 |
| 65 | 55 | Male | 1 | No | No | Yes | No | No | No | No | No | No | No | No | No | No | No | No | No |  | White British | Yes | Yes | Yes | Negative | 0/0 | Negative | 0/0 | Negative | 0 | 0 | 0 | 0 |
| 66 | 29 | Female | 2 | No | Yes | Yes | No | Yes | No | No | No | No | No | No | No | No | No | No | Yes | Coryza | White British | Yes | Yes | No | Negative | 0/0 | Negative | 0/0 | Negative | 0 | 0 | 0 | 0 |
| 67 | 47 | Male | 3 | No | Yes | No | No | No | No | No | No | No | No | No | No | No | No | No | No |  | Other | Yes | Yes | No | Negative | 0/0 | Negative | 0/0 | Negative | 0 | 0 | 0 | 0 |
| 68 | 37 | Female | 1 | No | Yes | No | No | No | No | No | No | No | No | No | No | No | No | No | No |  | White British | No | NA | NA | Negative | 0/0 | Negative | 0/0 | Negative | 0 | 0 | 0 | 0 |
| 69 | 27 | Female | 1 | No | Yes | No | No | No | No | No | No | No | No | No | No | No | No | No | No |  | White British | Yes | Yes | Yes | Negative | 0/0 | Negative | 0/0 | Negative | 0 | 0 | 0 | 0 |
| 70 | 38 | Female | 0 | No | Yes | No | No | No | No | No | No | No | No | No | No | No | No | No | No |  | Other | Yes | Yes | Yes | Negative | 0/0 | Negative | 0/0 | Negative | 0 | 0 | 0 | 0 |
| 71 | 32 | Female | 2 | No | Yes | No | No | No | No | No | No | No | No | No | No | No | No | No | No |  | Other | Yes | Yes | No | Negative | 0/0 | Negative | 0/0 | Negative | 0 | 0 | 0 | 0 |
| 72 | 30 | Female | 3 | No | Yes | No | No | No | No | No | No | No | No | No | No | No | No | No | No |  | White British | Yes | Yes | No | Negative | 0/0 | Negative | 0/0 | Negative | 0 | 0 | 0 | 0 |
| 73 | 23 | Male | 2 | No | Yes | No | No | No | No | No | No | No | No | No | No | No | No | No | Yes | Lost voice last week,runny nose | White British | No | NA | NA | Positive | 9/9 | Positive | 8/8 | Positive | 20.54 | 20.22 | 20.8 | 20.52 |
| 74 | 49 | Female | 2 | No | No | No | No | Yes | No | No | No | No | No | No | No | Yes | No | No | No |  | White British | Yes | Yes | Yes | Negative | 0/0 | Negative | 0/0 | Negative | 0 | 0 | 0 | 0 |
| 75 | 18 | Female | 7 | No | Yes | No | No | No | No | No | No | No | No | No | No | Yes | No | No | No |  | White British | No | NA | NA | Negative | 0/0 | Negative | 0/0 | Negative | 0 | 0 | 0 | 0 |
| 76 | 33 | Female | 0 | No | No | Yes | No | No | No | No | No | No | No | No | No | No | No | No | No |  | White British | Yes | Yes | No | Negative | 0/0 | Negative | 0/0 | Negative | 0 | 0 | 0 | 0 |
| 77 | 51 | Male | 3 | No | Yes | No | No | No | No | No | No | No | No | No | No | No | No | No | No |  | White British | Yes | Yes | No | Negative | 0/0 | Negative | 0/0 | Negative | 0 | 0 | 0 | 0 |
| 78 | 37 | Female | 5 | No | Yes | No | No | Yes | No | No | No | No | No | No | No | No | No | No | No |  | White British | No | NA | NA | Negative | 0/0 | Negative | 0/0 | Negative | 0 | 0 | 0 | 0 |
| 79 | 51 | Female | 2 | No | Yes | No | No | No | No | No | No | No | No | No | No | No | No | No | No |  | White British | Yes | Yes | Yes | Negative | 0/0 | Negative | 0/0 | Negative | 0 | 0 | 0 | 0 |
| 80 | 26 | Female | 2 | No | Yes | No | No | No | No | No | No | No | No | No | No | No | No | No | Yes | Coryza | White British | No | NA | NA | Negative | 0/0 | Negative | 0/0 | Negative | 0 | 0 | 0 | 0 |
| 81 | 37 | Female | 2 | No | Yes | No | No | No | No | No | No | No | No | No | No | No | No | No | No |  | White British | No | NA | NA | Negative | 0/0 | Negative | 0/0 | Negative | 0 | 0 | 0 | 0 |
| 82 | 40 | Female | 3 | No | Yes | No | No | No | No | No | No | No | No | No | No | No | No | No | No |  | White British | Yes | Yes | No | Negative | 0/0 | Negative | 0/0 | Negative | 0 | 0 | 0 | 0 |
| 83 | 38 | Male | 6 | No | No | Yes | No | No | No | No | No | No | Yes | No | Yes | No | No | No | No |  | Other | Yes | Yes | Yes | Negative | 0/0 | Negative | 0/0 | Negative | 0 | 0 | 0 | 0 |
| 84 | 30 | Female | 1 | No | Yes | Yes | No | Yes | No | No | No | No | No | No | No | No | No | No | Yes | Runny nose | White British | Yes | Yes | No | Negative | 0/0 | Negative | 0/0 | Negative | 0 | 0 | 0 | 0 |
| 85 | 34 | Female | 5 | No | Yes | No | No | No | No | No | No | No | No | No | No | No | No | No | Yes | Cold | White British | No | NA | NA | Negative | 0/0 | Negative | 0/0 | Negative | 0 | 0 | 0 | 0 |
| 86 | 34 | Female | 1 | No | Yes | No | No | No | No | No | No | No | No | No | No | No | No | No | No |  | Other | Yes | Yes | Yes | Negative | 0/0 | Negative | 0/0 | Negative | 0 | 0 | 0 | 0 |
| 87 | 30 | Female | 1 | No | No | Yes | No | No | No | No | No | No | No | No | No | No | No | No | No |  | White British | Yes | Yes | Yes | Negative | 0/0 | Negative | 0/0 | Negative | 0 | 0 | 0 | 0 |
| 88 | 37 | Female | 1 | No | Yes | No | No | No | No | No | No | No | No | No | No | No | No | No | No |  | White British | Yes | Yes | Yes | Negative | 0/0 | Negative | 0/0 | Negative | 0 | 0 | 0 | 0 |
| 89 | 50 | Male | 2 | No | No | No | No | No | No | No | No | Yes | No | No | No | No | No | No | No |  | White British | Yes | Yes | Yes | Negative | 0/0 | Negative | 0/0 | Negative | 0 | 0 | 0 | 0 |
| 90 | 32 | Male | 1 | No | Yes | No | No | No | No | No | No | No | No | No | No | Yes | No | No | No |  | White British | Yes | Yes | Yes | Negative | 0/0 | Negative | 0/0 | Negative | 0 | 0 | 0 | 0 |
| 91 | 69 | Female | 3 | No | Yes | No | No | Yes | No | No | No | No | No | No | No | Yes | No | No | Yes | Runny nose | White British | Yes | Yes | Yes | Negative | 0/0 | Negative | 0/0 | Negative | 0 | 0 | 0 | 0 |
| 92 | 58 | Male | 2 | No | Yes | No | No | No | No | No | No | No | No | No | No | No | No | No | No |  | White British | Yes | Yes | Yes | Negative | 0/0 | Negative | 0/0 | Negative | 0 | 0 | 0 | 0 |
| 93 | 35 | Female | 1 | No | Yes | No | No | No | No | No | No | No | No | No | No | No | No | No | No |  | White British | No | NA | NA | Negative | 0/0 | Negative | 0/0 | Negative | 0 | 0 | 0 | 0 |
| 94 | 29 | Female | 1 | No | Yes | No | No | No | No | No | No | No | No | No | No | No | No | No | No |  | White British | No | NA | NA | Negative | 0/0 | Negative | 0/0 | Negative | 0 | 0 | 0 | 0 |
| 95 | 68 | Female | 4 | No | Yes | No | No | No | No | No | No | No | No | No | No | No | No | No | No |  | White British | Yes | Yes | Yes | Negative | 0/0 | Negative | 0/0 | Negative | 0 | 0 | 0 | 0 |
| 96 | 49 | Female | 7 | No | Yes | No | No | No | No | No | No | No | No | No | No | No | No | No | No |  | White British | Yes | Yes | No | Negative | 0/0 | Negative | 0/0 | Negative | 0 | 0 | 0 | 0 |
| 97 | 42 | Female | 8 | No | No | Yes | No | No | No | No | No | No | Yes | Yes | Yes | Yes | No | No | Yes | Dizziness, blurred vision | Other | Yes | Yes | Yes | Negative | 0/0 | Negative | 0/0 | Negative | 0 | 0 | 0 | 0 |
| 98 | 54 | Female | 1 | No | Yes | No | No | No | No | No | No | No | No | No | No | No | No | No | No |  | White British | Yes | Yes | Yes | Negative | 0/0 | Negative | 0/0 | Negative | 0 | 0 | 0 | 0 |
| 99 | 59 | Female | 6 | No | No | No | No | Yes | No | No | No | No | Yes | No | Yes | No | No | No | Yes | Cold,runny nose | White British | Yes | Yes | No | Negative | 0/0 | Negative | 0/0 | Negative | 0 | 0 | 0 | 0 |
| 100 | 35 | Female | 2 | No | Yes | No | No | No | No | No | No | No | No | No | No | No | No | No | No |  | White British | Yes | Yes | No | Negative | 0/0 | Negative | 0/0 | Negative | 0 | 0 | 0 | 0 |
| 101 | 32 | Female | 2 | No | Yes | No | No | No | No | No | No | No | No | No | No | No | No | No | No |  | White British | Yes | Yes | No | Negative | 0/0 | Negative | 0/0 | Negative | 0 | 0 | 0 | 0 |
| 102 | 55 | Female | 5 | No | Yes | No | No | Yes | No | No | No | No | No | No | No | Yes | No | No | Yes | Ear ache , aches | White British | No | NA | NA | Positive | 9/9 | Positive | 10/10 | Positive | 25.78 | 25.45 | 25.86 | 25.69667 |
| 103 | 58 | Female | 0 | No | No | No | No | Yes | No | No | No | No | No | No | No | No | No | No | No |  | White British | Yes | Yes | Yes | Positive | 2/2 | Positive | 3/3 | Positive | 20.24 | 19.96 | 20.2 | 20.13333 |
| 104 | 42 | Male | 3 | No | Yes | No | Yes | No | No | No | No | No | No | No | No | Yes | No | No | Yes | Aches | White British | No | NA | NA | Positive | 10/10 | Positive | 9/9 | Positive | 16.98 | 16.49 | 16.99 | 16.82 |
| 105 | 48 | Male | 6 | No | No | No | No | No | No | No | No | No | No | No | No | No | No | No | Yes | Pneumonia | White British | Yes | Yes | Yes | Negative | 0/0 | Negative | 0/0 | Negative | 0 | 0 | 0 | 0 |
| 106 | 50 | Male | 5 | No | Yes | No | No | No | No | No | Yes | No | No | No | No | No | No | No | Yes | Runny nose/eyes,flu-like | Other | Yes | Yes | Yes | Positive | 3/3 | Negative | 0/0 | Positive | 21.63 | 21.41 | 22.01 | 21.68333 |
| 107 | 53 | Male | 1 | No | No | No | No | No | No | No | No | No | No | No | No | Yes | No | No | Yes | Achey all over | White British | Yes | Yes | Yes | Negative | 0/0 | Negative | 0/0 | Negative | 0 | 0 | 0 | 0 |
| 108 | 35 | Male | 4 | No | Yes | No | No | Yes | No | No | No | No | No | No | No | No | No | No | Yes | Sinus cold | White British | Yes | Yes | No | Negative | 0/0 | Negative | 0/0 | Negative | 0 | 0 | 0 | 0 |
| 109 | 25 | Male | 2 | No | Yes | No | No | No | No | No | No | No | No | No | No | No | No | No | No |  | White British | Yes | Yes | Yes | Negative | 0/0 | Negative | 0/0 | Negative | 0 | 0 | 0 | 0 |
| 110 | 53 | Female | 2 | No | Yes | No | No | No | No | No | No | No | No | No | No | No | No | No | No |  | White British | Yes | Yes | Yes | Negative | 0/0 | Negative | 0/0 | Negative | 0 | 0 | 0 | 0 |
| 111 | 63 | Female | 7 | No | Yes | No | No | No | No | No | No | No | No | No | No | No | No | No | No |  | White British | Yes | Yes | Yes | Negative | 0/0 | Negative | 0/0 | Negative | 0 | 0 | 0 | 0 |
| 112 | 62 | Female | 11 | No | Yes | No | No | No | No | No | No | No | No | No | No | No | No | No | No |  | White British | Yes | Yes | Yes | Negative | 0/0 | Negative | 0/0 | Negative | 0 | 0 | 0 | 0 |
| 113 | 19 | Female | 2 | No | No | No | No | Yes | No | No | No | No | No | No | No | No | No | No | Yes | Swollen glands | White British | Yes | Yes | Yes | Negative | 0/0 | Negative | 0/0 | Negative | 0 | 0 | 0 | 0 |
| 114 | 31 | Female | 2 | No | No | Yes | No | Yes | No | Yes | No | No | No | No | No | No | No | No | Yes | Aches, flu like symptoms | White British | Yes | Yes | No | Positive | 8/8 | Positive | 6/6 | Positive | 21.27 | 21.18 | 21.25 | 21.23333 |
| 115 | 20 | Male | 2 | No | No | No | No | No | No | No | No | No | No | Yes | Yes | No | No | No | Yes | Sweats | White British | No | NA | NA | Positive | 10/10 | Positive | 8/8 | Positive | 17.18 | 16.43 | 16.95 | 16.85333 |
| 116 | 57 | Male | 4 | No | Yes | Yes | No | No | No | No | Yes | Yes | No | No | No | Yes | No | No | No |  | White British | Yes | Yes | Yes | Positive | 2/2 | Positive | 3/3 | Positive | 27.76 | 27.29 | 27.8 | 27.61667 |
| 117 | 43 | Female | 1 | No | Yes | No | No | No | No | No | No | No | No | No | No | No | No | No | No |  | White British | Yes | Yes | Yes | Negative | 0/0 | Negative | 0/0 | Negative | 0 | 0 | 0 | 0 |
| 118 | 31 | Female | 3 | No | Yes | No | Yes | No | No | No | No | No | No | No | No | No | No | No | No |  | White British | Yes | Yes | Yes | Negative | 0/0 | Negative | 0/0 | Negative | 0 | 0 | 0 | 0 |
| 119 | 33 | Female | 2 | No | Yes | No | No | No | No | No | No | No | No | No | No | No | No | No | No |  | White British | Yes | Yes | Yes | Negative | 0/0 | Negative | 0/0 | Negative | 0 | 0 | 0 | 0 |
| 120 | 57 | Female | 3 | No | No | Yes | No | No | No | No | No | No | No | No | Yes | No | Yes | No | No |  | White British | Yes | Yes | Yes | Negative | 0/0 | Negative | 0/0 | Negative | 0 | 0 | 0 | 0 |
| 121 | 26 | Male | 2 | No | Yes | Yes | Yes | No | No | No | No | No | No | No | No | No | No | No | No |  | White British | Yes | Yes | No | Positive | 6/6 | Positive | 7/7 | Positive | 21.21 | 20.89 | 21.46 | 21.18667 |
| 122 | 41 | Male | 1 | No | No | Yes | No | No | No | No | No | No | No | No | No | No | No | No | No |  | White British | Yes | Yes | Yes | Negative | 0/0 | Negative | 0/0 | Negative | 0 | 0 | 0 | 0 |
| 123 | 23 | Female | 7 | No | Yes | No | No | No | No | No | Yes | Yes | No | No | No | No | No | No | No |  | White British | Yes | Yes | Yes | Negative | 0/0 | Negative | 0/0 | Positive | 29.45 | 29.27 | 29.71 | 29.47667 |
| 124 | 60 | Male | 3 | No | No | No | No | No | No | No | Yes | Yes | No | No | No | No | No | No | Yes | Aches | White British | Yes | Yes | Yes | Positive | 6/6 | Positive | 5/5 | Positive | 21 | 20.81 | 21.2 | 21.00333 |
| 125 | 34 | Male | 14 | No | Yes | No | No | No | No | No | No | No | No | No | No | No | No | No | No |  | White British | No | NA | NA | Negative | 0/0 | Negative | 0/0 | Negative | 0 | 0 | 0 | 0 |
| 126 | 28 | Male | 1 | No | No | Yes | No | Yes | No | No | No | No | No | No | No | No | No | No | No |  | White British | No | NA | NA | Negative | 0/0 | Negative | 0/0 | Negative | 0 | 0 | 0 | 0 |
| 127 | 34 | Male | 8 | No | Yes | Yes | No | No | No | No | Yes | Yes | No | No | No | No | Yes | No | No |  | White British | No | NA | NA | Positive | 10/10 | Positive | 6/6 | Positive | 20.13 | 19.78 | 20.62 | 20.17667 |
| 128 | 36 | Female | 2 | No | Yes | Yes | No | Yes | No | No | No | No | No | No | No | No | No | No | No |  | White British | Yes | Yes | Yes | Negative | 0/0 | Negative | 0/0 | Negative | 0 | 0 | 0 | 0 |
| 129 | 42 | Male | 2 | Yes | No | Yes | No | No | No | No | No | No | No | No | No | No | No | No | Yes | Aches | White British | No | NA | NA | Negative | 0/0 | Negative | 0/0 | Negative | 0 | 0 | 0 | 0 |
| 130 | 39 | Male | 4 | No | Yes | No | No | No | No | No | No | No | No | No | No | No | No | No | Yes | Tickley throat | White British | Yes | Yes | Yes | Negative | 0/0 | Negative | 0/0 | Negative | 0 | 0 | 0 | 0 |
| 131 | 42 | Male | 7 | No | Yes | No | No | No | No | No | No | No | No | No | No | No | No | No | Yes | Flu-like | White British | Yes | Yes | No | Positive | 3/3 | Negative | 0/0 | Positive | 27.93 | 27.75 | 28.3 | 27.99333 |
| 132 | 37 | Male | 4 | No | No | No | No | No | No | No | No | Yes | No | No | No | No | No | No | No |  | White British | Yes | Yes | Yes | Negative | 0/0 | Negative | 0/0 | Negative | 0 | 0 | 0 | 0 |
| 133 | 41 | Female | 14 | No | Yes | No | No | Yes | No | No | No | No | No | No | No | No | No | No | Yes | Runny nose | White British | Yes | Yes | No | Negative | 0/0 | Negative | 0/0 | Negative | 0 | 0 | 0 | 0 |
| 134 | 41 | Female | 2 | No | Yes | Yes | No | Yes | No | No | No | No | No | No | No | No | No | No | Yes | Chills,aches | White British | Yes | Yes | Yes | Positive | 9/9 | Positive | 2/2 | Positive | 19.57 | 18.99 | 19.41 | 19.32333 |
| 135 | 43 | Male | 1 | No | Yes | No | No | No | No | No | No | No | No | No | No | Yes | No | No | No |  | White British | Yes | Yes | Yes | Positive | 4/4 | Positive | 4/4 | Positive | 26.42 | 25.9 | 26.37 | 26.23 |
| 136 | 58 | Male | 0 | No | No | Yes | No | No | No | No | No | No | No | No | No | Yes | No | No | Yes | Runny nose,aches | White British | No | NA | NA | Positive | 5/5 | Positive | 7/7 | Positive | 17.65 | 17.43 | 17.86 | 17.64667 |
| 137 | 49 | Female | 2 | No | No | No | No | Yes | No | No | No | No | No | No | No | Yes | No | No | No |  | White British | Yes | Yes | Yes | Negative | 0/0 | Negative | 0/0 | Negative | 0 | 0 | 0 | 0 |
| 138 | 48 | Male | 1 | No | Yes | No | No | Yes | No | No | No | No | No | No | No | No | No | No | No |  | White British | Yes | Yes | Yes | Negative | 0/0 | Negative | 0/0 | Negative | 0 | 0 | 0 | 0 |
| 139 | 59 | Female | 4 | No | Yes | No | No | No | No | No | No | No | No | No | No | No | No | No | No |  | White British | Yes | Yes | Yes | Positive | 4/4 | Positive | 5/5 | Positive | 20.26 | 19.85 | 20.25 | 20.12 |
| 140 | 35 | Female | 3 | No | No | No | No | Yes | No | No | No | No | No | No | No | Yes | No | No | No |  | White British | Yes | Yes | No | Positive | 3/3 | Negative | 0/0 | Positive | 25.48 | 25.23 | 25.59 | 25.43333 |
| 141 | 33 | Female | 3 | No | Yes | No | No | No | No | No | Yes | Yes | No | No | No | No | No | No | No |  | White British | Yes | Yes | Yes | Negative | 0/0 | Negative | 0/0 | Negative | 0 | 0 | 0 | 0 |
| 142 | 47 | Female | 1 | No | No | Yes | No | No | No | No | Yes | Yes | No | No | No | No | No | No | Yes | aches | White British | Yes | Yes | Yes | Positive | 10/10 | Positive | 6/6 | Positive | 21.3 | 20.7 | 21.4 | 21.13333 |
| 143 | 32 | Male | 0 | No | Yes | No | No | Yes | No | No | No | No | No | No | Yes | No | Yes | No | No |  | White British | Yes | Yes | Yes | Negative | 0/0 | Negative | 0/0 | Negative | 0 | 0 | 0 | 0 |
| 144 | 24 | Female | 1 | No | Yes | No | No | No | No | No | No | No | No | No | No | No | No | No | No |  | White British | Yes | Yes | Yes | Positive | 9/9 | Positive | 4/4 | Positive | 23.26 | 22.97 | 23.62 | 23.28333 |
| 145 | 38 | Male | 1 | No | No | Yes | No | No | No | No | No | No | No | No | No | No | No | No | No |  | White British | No | NA | NA | Negative | 0/0 | Negative | 0/0 | Negative | 0 | 0 | 0 | 0 |
| 146 | 28 | Male | 1 | No | Yes | No | No | No | No | No | No | No | No | No | No | No | No | No | No |  | White British | Yes | Yes | No | Negative | 0/0 | Negative | 0/0 | Negative | 0 | 0 | 0 | 0 |
| 147 | 32 | Male | 1 | No | No | Yes | No | No | No | No | No | No | No | No | No | No | No | No | No |  | White British | Yes | Yes | Yes | Negative | 0/0 | Negative | 0/0 | Negative | 0 | 0 | 0 | 0 |
| 148 | 23 | Female | 1 | No | Yes | Yes | No | No | No | No | No | No | No | No | No | No | No | No | No |  | Other | Yes | Yes | No | Negative | 0/0 | Negative | 0/0 | Negative | 0 | 0 | 0 | 0 |
| 149 | 18 | Male | 2 | No | Yes | No | No | No | No | No | Yes | Yes | No | No | No | No | No | No | No |  | White British | Yes | Yes | No | Positive | 10/10 | Positive | 10/10 | Positive | 16.35 | 15.97 | 16.43 | 16.25 |
| 150 | 36 | Male | 3 | No | Yes | No | No | No | No | No | No | No | No | No | No | No | No | No | No |  | Other | Yes | Yes | Yes | Negative | 0/0 | Negative | 0/0 | Negative | 0 | 0 | 0 | 0 |
| 151 | 37 | Male | 0 | No | No | No | No | Yes | No | No | No | No | No | No | No | No | No | No | No |  | White British | No | NA | NA | Negative | 0/0 | Negative | 0/0 | Negative | 0 | 0 | 0 | 0 |
| 152 | 34 | Female | 2 | No | Yes | Yes | No | Yes | No | No | No | No | No | No | No | Yes | Yes | No | No |  | White British | No | NA | NA | Negative | 0/0 | Negative | 0/0 | Positive | 33.1 | 33.26 | 33.11 | 33.15667 |
| 153 | 52 | Male | 1 | No | Yes | Yes | No | No | No | No | No | No | No | No | No | No | No | No | No |  | White British | Yes | Yes | Yes | Positive | 8/8 | Positive | 6/6 | Positive | 19.68 | 19.05 | 19.29 | 19.34 |
| 154 | 21 | Female | 3 | Yes | No | No | Yes | No | No | No | No | No | No | No | No | Yes | No | No | No |  | White British | Yes | Yes | No | Negative | 0/0 | Negative | 0/0 | Negative | 0 | 0 | 0 | 0 |
| 155 | 82 | Male | 1 | No | Yes | Yes | No | No | No | No | No | No | No | No | No | No | No | No | No |  | White British | Yes | Yes | Yes | Negative | 0/0 | Negative | 0/0 | Negative | 0 | 0 | 0 | 0 |
| 156 | 21 | Female | 1 | No | Yes | No | No | No | No | No | Yes | Yes | No | No | No | Yes | No | No | Yes | Tender skin | White British | No | NA | NA | Positive | 10/10 | Positive | 10/10 | Positive | 16.8 | 16.69 | 16.95 | 16.81333 |
| 157 | 20 | Female | 1 | Yes | Yes | No | No | No | No | No | Yes | Yes | No | No | No | Yes | No | No | No |  | White British | No | NA | NA | Positive | 10/10 | Positive | 10/10 | Positive | 17.18 | 16.76 | 17.11 | 17.01667 |
| 158 | 37 | Male | 0 | No | Yes | No | No | No | No | No | No | No | No | No | No | No | No | No | No |  | White British | Yes | Yes | Yes | Negative | 0/0 | Negative | 0/0 | Negative | 0 | 0 | 0 | 0 |
| 159 | 50 | Male | 1 | No | Yes | Yes | No | No | No | No | No | No | No | No | No | No | No | No | No |  | Other | No | NA | NA | Positive | 8/8 | Positive | 10/10 | Positive | 20.75 | 20.45 | 21.05 | 20.75 |
| 160 | 34 | Male | 2 | No | No | No | No | Yes | No | No | No | No | No | No | No | No | No | No | No |  | White British | Yes | Yes | Yes | Negative | 0/0 | Negative | 0/0 | Negative | 0 | 0 | 0 | 0 |
| 161 | 28 | Female | 8 | No | Yes | Yes | No | No | No | No | Yes | Yes | No | No | No | No | No | No | No |  | White British | Yes | Yes | No | Negative | 0/0 | Negative | 0/0 | Negative | 0 | 0 | 0 | 0 |
| 162 | 40 | Female | 1 | No | No | Yes | No | Yes | No | No | No | No | No | No | No | No | No | No | No |  | White British | Yes | Yes | Yes | Negative | 0/0 | Negative | 0/0 | Negative | 0 | 0 | 0 | 0 |
| 163 | 54 | Male | 2 | Yes | Yes | Yes | No | Yes | No | No | Yes | Yes | No | No | No | Yes | No | No | No |  | White British | Yes | Yes | Yes | Positive | 8/8 | Positive | 9/9 | Positive | 21.62 | 21.47 | 21.6 | 21.56333 |
| 164 | 31 | Female | 0 | No | Yes | Yes | No | Yes | No | No | No | No | No | No | No | Yes | No | No | No |  | White British | Yes | Yes | Yes | Negative | 0/0 | Negative | 0/0 | Negative | 0 | 0 | 0 | 0 |
| 165 | 36 | Male | 2 | No | Yes | Yes | No | No | No | No | No | No | No | No | No | No | No | No | Yes | Body aches | Other | Yes | Yes | No | Positive | 7/7 | Positive | 3/3 | Positive | 21.2 | 20.75 | 21.43 | 21.12667 |
| 166 | 25 | Female | 0 | No | No | No | No | No | No | No | No | No | No | No | No | Yes | Yes | No | Yes | Runny nose | White British | Yes | Yes | No | Positive | I/I | Positive | 4/4 | Positive | 21.28 | 20.75 | 20.87 | 20.96667 |
| 167 | 22 | Female | 1 | No | Yes | No | No | Yes | No | No | No | No | No | No | No | No | No | No | No |  | White British | Yes | Yes | No | Negative | 0/0 | Negative | 0/0 | Negative | 0 | 0 | 0 | 0 |
| 168 | 22 | Male | 1 | No | Yes | Yes | No | No | No | No | No | No | No | No | No | Yes | No | No | No |  | White British | Yes | Yes | No | Positive | 10/10 | Positive | 8/8 | Positive | 16.04 | 15.62 | 16.24 | 15.96667 |
| 169 | 18 | Male | 3 | No | No | No | No | No | No | No | Yes | Yes | No | No | No | No | No | No | No |  | White British | No | NA | NA | Positive | 2/2 | Negative | 0/0 | Positive | 28.04 | 28.11 | 28.13 | 28.09333 |
| 170 | 18 | Female | 4 | No | Yes | No | Yes | No | No | No | No | Yes | No | Yes | No | Yes | No | No | No |  | White British | Yes | Yes | No | Negative | 0/0 | Negative | 0/0 | Positive | 30.2 | 30.1 | 29.76 | 30.02 |
| 171 | 23 | Female | 4 | No | Yes | Yes | No | Yes | No | No | No | No | Yes | No | No | No | No | No | No |  | White British | Yes | Yes | No | Negative | 0/0 | Negative | 0/0 | Negative | 0 | 0 | 0 | 0 |
| 172 | 25 | Male | 8 | No | Yes | No | Yes | Yes | No | No | No | No | No | No | Yes | Yes | No | No | No |  | White British | Yes | Yes | No | Negative | 0/0 | Negative | 0/0 | Positive | 28.03 | 27.53 | 27.83 | 27.79667 |
| 173 | 25 | Male | 2 | No | Yes | No | No | No | No | No | No | No | No | No | No | No | No | No | No |  | White British | Yes | Yes | No | Negative | 0/0 | Negative | 0/0 | Negative | 0 | 0 | 0 | 0 |
| 174 | 57 | Female | 3 | No | Yes | No | No | No | No | No | No | No | No | No | No | No | No | No | No |  | White British | Yes | Yes | Yes | Negative | 0/0 | Negative | 0/0 | Negative | 0 | 0 | 0 | 0 |
| 175 | 46 | Female | 1 | No | Yes | No | No | No | No | No | No | No | No | No | No | No | No | Yes | No |  | White British | Yes | Yes | Yes | Negative | 0/0 | Negative | 0/0 | Negative | 0 | 0 | 0 | 0 |
| 176 | 21 | Male | 3 | No | Yes | No | No | Yes | No | No | No | No | No | No | No | No | No | No | Yes | runny nose | Other | Yes | Yes | No | Negative | 0/0 | Negative | 0/0 | Negative | 0 | 0 | 0 | 0 |
| 177 | 49 | Female | 1 | No | No | Yes | No | Yes | No | No | No | No | No | No | No | Yes | No | No | No |  | White British | Yes | Yes | Yes | Negative | 0/0 | Negative | 0/0 | Negative | 0 | 0 | 0 | 0 |
| 178 | 40 | Male | 3 | No | Yes | No | No | Yes | No | No | No | No | No | No | No | Yes | No | No | No |  | Other | No | NA | NA | Negative | 0/0 | Negative | 0/0 | Negative | 0 | 0 | 0 | 0 |
| 179 | 25 | Female | 0 | No | Yes | No | No | No | No | No | No | No | No | No | No | No | No | No | No |  | White British | Yes | Yes | No | Negative | 0/0 | Negative | 0/0 | Negative | 0 | 0 | 0 | 0 |
| 180 | 20 | Male | 1 | No | Yes | Yes | No | No | No | No | No | No | No | No | No | No | No | No | No |  | White British | Yes | Yes | No | Positive | 5/5 | Positive | 2/2 | Positive | 19.84 | 19.48 | 19.96 | 19.76 |
| 181 | 39 | Female | 1 | No | Yes | No | Yes | No | No | No | Yes | No | No | No | No | No | No | No | No |  | White British | Yes | Yes | Yes | Negative | 0/0 | Negative | 0/0 | Negative | 0 | 0 | 0 | 0 |
| 182 | 38 | Male | 3 | No | Yes | No | No | No | No | No | No | No | No | No | No | No | No | No | Yes | pain, flu symptoms | White British | No | NA | NA | Positive | 9/9 | Positive | 5/5 | Positive | 19.66 | 18.79 | 19.49 | 19.31333 |
| 183 | 71 | Female | 10 | No | Yes | No | No | No | No | No | No | No | No | No | No | No | No | No | No |  | White British | Yes | Yes | Yes | Negative | 0/0 | Negative | 0/0 | Negative | 0 | 0 | 0 | 0 |
| 184 | 31 | Female | 2 | No | Yes | Yes | No | No | No | No | No | No | No | No | No | No | No | No | No |  | Other | Yes | Yes | Yes | Negative | 0/0 | Negative | 0/0 | Negative | 0 | 0 | 0 | 0 |
| 185 | 28 | Female | 1 | No | No | Yes | No | No | No | No | Yes | No | No | No | No | No | No | No | No |  | White British | Yes | Yes | Yes | Negative | 0/0 | Negative | 0/0 | Negative | 0 | 0 | 0 | 0 |
| 186 | 66 | Male | 1 | No | No | No | No | No | No | No | Yes | Yes | No | No | No | No | No | No | Yes | Flu symptoms | White British | Yes | Yes | Yes | Positive | 6/6 | Positive | 10/10 | Positive | 18.94 | 18.59 | 18.72 | 18.75 |
| 187 | 33 | Male | 0 | No | Yes | No | No | Yes | No | No | No | No | No | No | No | No | No | No | No |  | White British | No | NA | NA | Positive | 4/4 | Positive | 2/2 | Positive | 24.72 | 24.62 | 24.61 | 24.65 |
| 188 | 63 | Male | 3 | No | No | No | No | No | No | No | Yes | No | No | No | No | No | No | No | No |  | White British | Yes | Yes | Yes | Negative | 0/0 | Negative | 0/0 | Negative | 0 | 0 | 0 | 0 |
| 189 | 18 | Female | 0 | No | No | No | No | Yes | No | No | No | No | No | No | No | No | No | No | Yes | Runny nose | Other | Yes | Yes | No | Positive | 8/8 | Negative | 0/0 | Positive | 20.3 | 20.34 | 20.41 | 20.35 |
| 190 | 33 | Female | 0 | No | No | No | No | No | No | No | Yes | Yes | No | No | No | No | No | No | No |  | White British | Yes | Yes | No | Negative | 0/0 | Negative | 0/0 | Negative | 0 | 0 | 0 | 0 |
| 191 | 28 | Female | 2 | No | Yes | No | No | No | No | No | No | No | No | No | No | No | No | No | No |  | White British | Yes | Yes | Yes | Negative | 0/0 | Negative | 0/0 | Negative | 0 | 0 | 0 | 0 |
| 192 | 56 | Female | 1 | No | No | Yes | No | No | No | No | No | No | No | No | No | No | No | No | No |  | White British | Yes | Yes | Yes | Positive | 10/10 | Positive | 9/9 | Positive | 22.44 | 22.2 | 22.75 | 22.46333 |
| 193 | 55 | Female | 3 | No | No | No | No | No | No | No | No | No | No | No | No | Yes | No | No | Yes | hot and cold | White British | Yes | Yes | Yes | Positive | 5/5 | Positive | 6/6 | Positive | 25.42 | 25.31 | 25.6 | 25.44333 |
| 194 | 63 | Male | 1 | No | Yes | No | No | No | No | No | No | No | No | No | No | No | No | No | Yes | sweating | White British | Yes | Yes | Yes | Positive | 10/10 | Positive | 9/9 | Positive | 17.13 | 17.01 | 17.24 | 17.12667 |
| 195 | 19 | Male | 1 | No | Yes | No | No | No | No | No | No | No | No | No | No | No | No | No | No |  | White British | Yes | Yes | No | Positive | 3/3 | Negative | 0/0 | Positive | 22.99 | 22.88 | 23.01 | 22.96 |
| 196 | 25 | Female | 0 | No | Yes | No | No | No | No | No | No | No | No | No | No | No | No | No | Yes | blocked nose | White British | Yes | Yes | Yes | Positive | 8/8 | Positive | 5/5 | Positive | 17.81 | 17.66 | 17.99 | 17.82 |
| 197 | 22 | Male | 1 | No | No | No | No | No | No | No | Yes | Yes | No | No | No | No | No | No | No |  | White British | No | NA | NA | Positive | 6/6 | Positive | 4/4 | Positive | 17.61 | 18.04 | 18.31 | 17.98667 |
| 198 | 60 | Female | 1 | No | Yes | No | No | Yes | No | No | No | No | No | No | No | No | No | No | Yes | runny nose | White British | Yes | Yes | Yes | Negative | 0/0 | Negative | 0/0 | Negative | 0 | 0 | 0 | 0 |
| 199 | 38 | Male | 1 | No | Yes | Yes | No | No | No | No | No | No | No | No | No | No | No | No | No |  | White British | No | NA | NA | Negative | 0/0 | Negative | 0/0 | Positive | 20.59 | 19.83 | 20.4 | 20.27333 |
| 200 | 53 | Male | 2 | No | Yes | No | No | Yes | No | No | No | No | No | No | No | No | No | No | No |  | White British | Yes | Yes | Yes | Negative | 0/0 | Negative | 0/0 | Negative | 0 | 0 | 0 | 0 |
| 201 | 26 | Male | 11 | No | No | No | No | No | No | No | Yes | Yes | No | No | No | No | No | No | No |  | White British | No | NA | NA | Negative | 0/0 | Negative | 0/0 | Positive | 25.63 | 25.67 | 26.07 | 25.79 |
| 202 | 31 | Female | 1 | No | Yes | No | No | No | No | No | No | No | No | No | No | No | No | No | No |  | White British | Yes | Yes | Yes | Negative | 0/0 | Negative | 0/0 | Negative | 0 | 0 | 0 | 0 |
| 203 | 32 | Male | 3 | No | Yes | Yes | No | Yes | No | No | No | No | No | No | No | No | No | No | No |  | White British | Yes | NA | NA | Positive | 8/8 | Positive | 10/10 | Positive | 15.91 | 15.25 | 15.99 | 15.71667 |
| 204 | 50 | Male | 3 | No | No | Yes | No | No | No | No | No | No | No | No | No | No | No | No | No |  | White British | Yes | Yes | Yes | Positive | 6/6 | Positive | 2/2 | Positive | 20.5 | 19.74 | 20.23 | 20.15667 |
| 205 | 60 | Female | 4 | No | Yes | No | No | No | No | No | No | No | No | No | No | No | No | No | No |  | Other | Yes | Yes | Yes | Negative | 0/0 | Negative | 0/0 | Negative | 0 | 0 | 0 | 0 |
| 206 | 66 | Female | 2 | No | Yes | Yes | No | No | No | No | No | No | No | No | No | No | No | No | Yes | aches | White British | Yes | Yes | Yes | Positive | 10/10 | Positive | 6/6 | Positive | 18.8 | 18.35 | 18.75 | 18.63333 |
| 207 | 41 | Male | 1 | No | No | Yes | No | No | No | No | No | No | No | No | No | No | No | No | No |  | White British | Yes | Yes | Yes | Positive | 3/3 | Positive | 9/9 | Positive | 19.66 | 19.27 | 19.99 | 19.64 |
| 208 | 42 | Female | 2 | No | Yes | Yes | No | No | No | No | No | No | No | No | No | No | No | No | No |  | White British | Yes | Yes | Yes | Positive | 3/3 | Positive | 3/3 | Positive | 19.2 | 18.49 | 18.95 | 18.88 |
| 209 | 39 | Male | 1 | No | Yes | Yes | No | Yes | No | No | No | No | No | No | No | No | No | No | No |  | White British | Yes | Yes | Yes | Positive | 4/4 | Positive | 6/6 | Positive | 18.64 | 17.82 | 18.62 | 18.36 |
| 210 | 21 | Female | 1 | No | Yes | Yes | No | No | No | No | No | No | No | No | No | No | No | No | No |  | White British | Yes | Yes | No | Positive | 7/7 | Positive | 10/10 | Positive | 17.44 | 16.81 | 17.26 | 17.17 |
| 211 | 51 | Female | 1 | No | Yes | No | Yes | No | No | No | No | No | No | No | No | No | No | No | No |  | White British | No | NA | NA | Positive | 3/3 | Positive | 6/6 | Positive | 20.06 | 19.39 | 19.94 | 19.79667 |
| 212 | 59 | Female | 1 | No | Yes | No | No | No | No | No | No | No | No | No | Yes | No | No | No | No |  | Other | Yes | Yes | Yes | Negative | 0/0 | Negative | 0/0 | Negative | 0 | 0 | 0 | 0 |
| 213 | 22 | Female | 2 | No | Yes | No | No | No | No | No | No | No | No | No | No | Yes | No | No | No |  | White British | No | NA | NA | Positive | 10/10 | Positive | 5/5 | Positive | 17.5 | 17.02 | 17.64 | 17.38667 |
| 214 | 31 | Female | 1 | No | Yes | No | No | No | No | No | No | No | No | No | No | Yes | No | No | No |  | White British | Yes | Yes | Yes | Negative | 0/0 | Negative | 0/0 | Positive | 37.97 | 37.83 | 38.62 | 38.14 |
| 215 | 25 | Female | 1 | No | No | Yes | No | Yes | No | No | No | No | No | No | No | No | No | No | Yes | aches | White British | Yes | Yes | Yes | Positive | 4/4 | Positive | 9/9 | Positive | 19.59 | 18.78 | 19.34 | 19.23667 |
| 216 | 31 | Female | 1 | No | Yes | No | No | No | No | No | Yes | No | No | No | No | No | No | No | Yes | cold | Other | Yes | Yes | Yes | Positive | 3/3 | Positive | 8/8 | Positive | 21.3 | 20.35 | 20.63 | 20.76 |
| 217 | 44 | Female | 2 | No | Yes | No | No | No | No | No | No | No | No | No | No | Yes | No | No | No |  | Other | Yes | Yes | Yes | Positive | 2/2 | Positive | 3/3 | Positive | 30.97 | 30.5 | 30.84 | 30.77 |
| 218 | 22 | Male | 2 | No | Yes | No | No | No | No | No | Yes | Yes | No | No | No | No | No | No | No |  | White British | Yes | Yes | No | Positive | 3/3 | Positive | 7/7 | Positive | 18.45 | 17.92 | 18.57 | 18.31333 |
| 219 | 29 | Male | 32 | No | No | No | No | Yes | No | No | No | No | No | No | No | No | No | No | No |  | White British | No | NA | NA | Positive | 8/8 | Positive | 2/2 | Positive | 29.83 | 29.13 | 29.53 | 29.49667 |
| 220 | 45 | Male | 1 | No | No | No | No | No | No | No | No | No | No | No | No | No | No | No | Yes | aches/flu symptom | White British | Yes | Yes | Yes | Negative | 0/0 | Negative | 0/0 | Negative | 0 | 0 | 0 | 0 |
| 221 | 40 | Male | 3 | No | No | Yes | Yes | No | No | No | No | No | Yes | No | No | No | No | No | Yes | aches | White British | Yes | Yes | Yes | Positive | 10/10 | Positive | 6/6 | Positive | 22.06 | 21.22 | 21.93 | 21.73667 |
| 222 | 21 | Male | 3 | No | Yes | Yes | No | No | No | No | No | No | No | No | No | Yes | No | No | No |  | White British | Yes | NA | NA | Positive | 8/8 | Positive | 5/5 | Positive | 20.31 | 20.59 | 20.96 | 20.62 |
| 223 | 30 | Female | 2 | No | Yes | No | No | Yes | No | No | No | No | No | No | No | No | No | No | No |  | White British | Yes | Yes | No | Negative | 0/0 | Negative | 0/0 | Negative | 0 | 0 | 0 | 0 |
| 224 | 37 | Female | 0 | No | Yes | Yes | No | No | No | No | No | No | No | No | No | No | No | No | No |  | Other | Yes | Yes | Yes | Negative | 0/0 | Negative | 0/0 | Negative | 0 | 0 | 0 | 0 |
| 225 | 19 | Male | 0 | No | No | No | No | Yes | No | No | No | No | No | No | No | No | No | No | Yes | runny nose | White British | No | NA | NA | Positive | 10/10 | Positive | 10/10 | Positive | 16.14 | 15.26 | 16.08 | 15.82667 |
| 226 | 38 | Female | 1 | No | No | No | No | No | No | No | No | No | No | Yes | Yes | No | No | No | Yes | aches | White British | Yes | Yes | Yes | Negative | 0/0 | Negative | 0/0 | Negative | 0 | 0 | 0 | 0 |
| 227 | 65 | Female | 3 | No | Yes | Yes | No | No | No | No | No | No | No | No | No | No | No | No | No |  | White British | Yes | Yes | Yes | Negative | 0/0 | Negative | 0/0 | Negative | 0 | 0 | 0 | 0 |
| 228 | 68 | Male | 3 | No | Yes | Yes | No | No | No | No | No | No | No | No | No | No | No | No | No |  | White British | Yes | Yes | Yes | Negative | 0/0 | Negative | 0/0 | Negative | 0 | 0 | 0 | 0 |
| 229 | 25 | Male | 1 | No | Yes | Yes | No | Yes | No | No | No | No | No | No | No | No | Yes | No | Yes | sore eyes | Other | Yes | Yes | No | Positive | 10/10 | Positive | 9/9 | Positive | 18.14 | 17.64 | 17.95 | 17.91 |
| 230 | 47 | Female | 2 | No | Yes | No | No | Yes | No | No | No | No | No | No | No | Yes | No | No | No |  | White British | Yes | Yes | Yes | Negative | 0/0 | Negative | 0/0 | Negative | 0 | 0 | 0 | 0 |
| 231 | 41 | Male | 1 | No | Yes | No | No | Yes | No | No | No | No | No | No | No | No | No | No | No |  | White British | Yes | Yes | Yes | Positive | 6/6 | Negative | 0/0 | Positive | 27.74 | 26.87 | 27.39 | 27.33333 |
| 232 | 44 | Female | 1 | No | No | No | No | Yes | No | No | No | No | No | No | No | Yes | No | No | No |  | White British | Yes | Yes | Yes | Negative | 0/0 | Negative | 0/0 | Negative | 0 | 0 | 0 | 0 |
| 233 | 47 | Male | 0 | No | No | No | No | No | No | No | No | No | No | No | No | Yes | No | No | No |  | White British | Yes | Yes | Yes | Negative | 0/0 | Negative | 0/0 | Negative | 0 | 0 | 0 | 0 |
| 234 | 35 | Male | 4 | No | No | No | No | Yes | No | No | Yes | No | No | No | No | Yes | No | No | Yes | dizziness | White British | Yes | Yes | Yes | Negative | 0/0 | Negative | 0/0 | Negative | 0 | 0 | 0 | 0 |
| 235 | 49 | Female | 1 | No | Yes | No | No | Yes | No | No | No | No | No | No | No | No | No | No | No |  | White British | Yes | Yes | Yes | Positive | 9/9 | Positive | 7/7 | Positive | 17.04 | 16.06 | 16.45 | 16.51667 |
| 236 | 60 | Male | 2 | No | Yes | No | No | Yes | No | No | No | No | No | No | No | No | No | No | Yes | runny nose | White British | Yes | Yes | Yes | Negative | 0/0 | Negative | 0/0 | Negative | 0 | 0 | 0 | 0 |
| 237 | 47 | Female | 2 | No | No | Yes | No | No | No | No | No | No | No | No | No | No | No | No | No |  | Other | Yes | Yes | Yes | Negative | 0/0 | Negative | 0/0 | Negative | 0 | 0 | 0 | 0 |
| 238 | 22 | Female | 2 | No | No | No | No | Yes | No | No | No | No | No | No | No | Yes | No | No | Yes | runny nose | White British | Yes | Yes | Yes | Negative | 0/0 | Negative | 0/0 | Negative | 0 | 0 | 0 | 0 |
| 239 | 52 | Female | 1 | No | No | Yes | No | No | No | No | No | No | No | No | No | No | Yes | No | No |  | White British | Yes | Yes | Yes | Negative | 0/0 | Negative | 0/0 | Negative | 0 | 0 | 0 | 0 |
| 240 | 42 | Female | 1 | No | No | Yes | No | Yes | No | No | No | No | No | No | No | Yes | No | No | No |  | White British | Yes | Yes | Yes | Negative | 0/0 | Negative | 0/0 | Negative | 0 | 0 | 0 | 0 |
| 241 | 60 | Female | 2 | No | Yes | No | No | No | No | No | No | No | No | No | No | No | No | No | No |  | White British | Yes | Yes | Yes | Negative | 0/0 | Negative | 0/0 | Negative | 0 | 0 | 0 | 0 |
| 242 | 33 | Male | 0 | No | No | No | No | Yes | No | No | No | No | No | No | No | No | No | No | Yes | Blocked nose | Other | Yes | Yes | Yes | Negative | 0/0 | Negative | 0/0 | Negative | 0 | 0 | 0 | 0 |
| 243 | 72 | Male | 7 | No | Yes | Yes | No | No | No | No | No | No | No | No | No | No | No | No | No |  | White British | Yes | Yes | Yes | Negative | 0/0 | Negative | 0/0 | Negative | 0 | 0 | 0 | 0 |
| 244 | 25 | Female | 3 | No | Yes | No | No | No | No | No | No | No | No | Yes | No | No | No | No | Yes | Ear,Jaw,Throat pain | White British | Yes | Yes | Yes | Negative | 0/0 | Negative | 0/0 | Negative | 0 | 0 | 0 | 0 |
| 245 | 70 | Male | 1 | No | Yes | Yes | No | No | No | No | No | No | No | No | No | No | No | No | No |  | White British | Yes | Yes | Yes | Positive | 10/10 | Positive | 3/3 | Positive | 15.28 | 15.03 | 15.47 | 15.26 |
| 246 | 42 | Female | 1 | No | Yes | No | No | No | No | No | Yes | Yes | No | No | No | No | No | No | No |  | White British | Yes | Yes | Yes | Positive | 5/5 | Positive | 9/9 | Positive | 22.59 | 22.25 | 22.81 | 22.55 |
| 247 | 26 | Female | 2 | No | Yes | No | No | No | No | No | No | No | No | No | No | No | No | No | No |  | White British | Yes | Yes | Yes | Negative | 0/0 | Negative | 0/0 | Negative | 0 | 0 | 0 | 0 |
| 248 | 26 | Female | 1 | No | Yes | No | No | Yes | No | No | No | No | No | No | No | No | No | No | Yes | sinus pressure | White British | Yes | Yes | No | Positive | 7/7 | Positive | 7/7 | Positive | 25.88 | 25.18 | 25.43 | 25.49667 |
| 249 | 45 | Female | 4 | No | No | No | No | No | No | No | No | No | No | Yes | Yes | No | Yes | No | No |  | White British | Yes | Yes | Yes | Positive | 5/5 | Negative | 0/0 | Positive | 28.16 | 27.47 | 27.96 | 27.86333 |
