# Supplementary figures and images for: A prospective diagnostic evaluation of accuracy of self-taken and healthcare worker-taken swabs for rapid COVID-19 testing
Source: PLoS One. 2022 Jun 30;17(6):e0270715. doi: 10.1371/journal.pone.0270715 (PMC9246218; doi:10.1371/journal.pone.0270715)

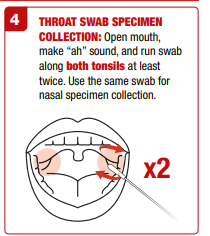

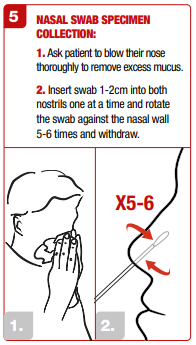

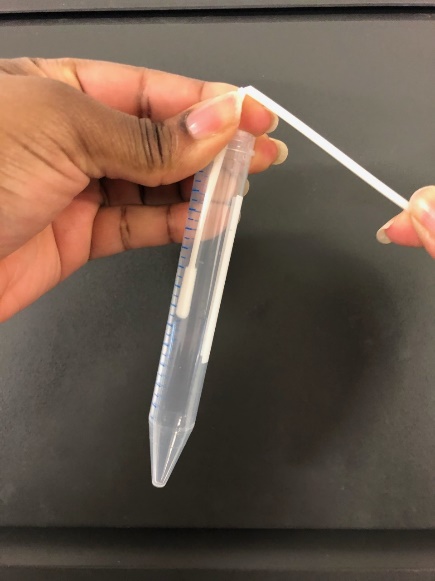


Put in green tube and snap swab in half. Screw up lid.

Supplement: S1 File — (DOCX) [file pone.0270715.s003.docx]
