# Supplementary material for: A prospective diagnostic evaluation of accuracy of self-taken and healthcare worker-taken swabs for rapid COVID-19 testing
Source: PLoS One. 2022 Jun 30;17(6):e0270715. doi: 10.1371/journal.pone.0270715 (PMC9246218; doi:10.1371/journal.pone.0270715)
Supplement: Attachment — Submitted filename: Response to reviewers.docx [file pone.0270715.s005.docx]

Department of Clinical Sciences,

Pembroke Place,

Liverpool,

L3 5QA.

To

Sylvia Maria Bruisten, Ph.D

Academic Editor

PLOS ONE

10^th^ June 2022

Revised Manuscript: PONE-D-22-05402R1

Dear Editor,

We would like to thank the editor for their constructive comments and recommendations made to this manuscript. We have reviewed the feedback from yourself and Reviewer 2 and have made the revisions suggested and have prepared an itemized reply to all issues raised on the following pages as requested.

We have especially focused on the comments and recommendations from Reviewer 2 and have reviewed our data to address their concerns. We have ensured that the subset of participants does not change the outcome or message of the paper and made this much clearer in the limitations section of our work. We hope that the addition of this information will be satisfactory and have also included their suggestions for further work which we are sadly unable to carry out as we do not have the scope or resources to undertake additional experiments at this time.

We hope these replies are satisfactory to the reviewers and your office.

We look forward to your decision.

Best regards,

Helen Savage

On behalf of all authors.

PONE-D-22-05402R1

A prospective diagnostic evaluation of accuracy of self-taken and healthcare worker-taken swabs for rapid COVID-19 testing

**Response to comments from Editor**

Since only one of the previous reviewers was available to look at the rebuttal I have now also looked at it as reviewer number 4. There is still a comment on the lack of (internal) controls and the small group of samples with relatively high Ct values, which have a large influence on the results. Please clarify if this has an impact on the main message of your paper.

Reviewer 2 still has a problem with the comment that there is a large influence of the group of samples with Ct values ranging between 25 and 30 and that this is a small group. If possible you should further discuss why you think that the data are still sound. Please make more clear that this will not influence the message of the paper.

Thank you for these helpful comments, and we will respond to the comments from the Editor and from Reviewer to together here.

We have carefully rereviewed the data in the manuscript and revised the wording of the text in the Discussion section to make clear that the subset of data would not change the overall conclusion of the paper if excluded and further work that could be undertaken to characterise this group.

The revised text (Line 265 – 279) now reads:

The majority of participants with discrepant results between self-taken and HCW taken RDT results had a mean CT of between 25-30 on PCR (six of ten). Although there was only a small number of participants with a CT value in this range, these values are influential in estimates of the sensitivity of the HCW taken RDT tests. Because of the small numbers, we did not undertake further formal statistical analysis; however when participants with CT values within this range were excluded, our main conclusion that self-taken swabs were non-inferior to healthcare worker taken swabs did not change (sensitivity with CT 25-29.9 excluded: self taken RDT 91.7% [55/60]; HCW taken 88.3% [53/55]). Similar results were noted in the trial by García-Fiñana et al4 who also saw a decrease in sensitivity of RDTs compared to PCR in samples with a CT of greater than 24.4. Future research could compare self-taken and healthcare worker-taken samples by PCR including housekeeper genes to see if the quality of sampling varies and may affect CT values, particularly for samples with CT values over 25, as lower sampling quality could result in lower CT values and therefore a perceived poorer performance in this range.

**Response to Comments from Reviewer 2**

In my opinion the comments are addressed only minor, and despite the conclusions are discussed in a better way, the paper still lacks the proper controls/control experiments supporting the differences shown driven by the group with PCR positivity 25-29.

Thank you for the suggestion. Please additionally see the Response to the Editor’s comments above where we have aimed to make it clear that this small group does not affect the conclusions of the paper. The PCR used was a WHO approved PCR and therefore we were not able to make alterations to it but we also agree this is an interesting idea and we believe would make a complementary piece of follow-up work using an in-house PCR and have therefore included this within the limitations as a piece of additional experimentation that would be beneficial as future research.
